# A Pilot Clinical Trial to Objectively Assess the Efficacy of Electroacupuncture on Gait in Patients with Parkinson's Disease Using Body Worn Sensors

**DOI:** 10.1371/journal.pone.0155613

**Published:** 2016-05-26

**Authors:** Hong Lei, Nima Toosizadeh, Michael Schwenk, Scott Sherman, Stephan Karp, Esther Sternberg, Bijan Najafi

**Affiliations:** 1 Department of Neurology, College of Medicine, University of Arizona, Tucson, Arizona, United States of America; 2 Interdisciplinary Consortium on Advanced Motion Performance (iCAMP) and Southern Arizona Limb Salvage Alliance (SALSA), Department of Surgery, College of Medicine, University of Arizona, Tucson, Arizona, United States of America; 3 Arizona Center on Aging, University of Arizona, Tucson, Arizona, United States of America; 4 Arizona Center for Integrative Medicine, University of Arizona, Tucson, Arizona, United States of America; 5 Michael E. DeBakey Department of Surgery, Baylor College of Medicine, Houston, Texas, United States of America; St Francis Hospital, UNITED STATES

## Abstract

**Background:**

Gait disorder, a key contributor to fall and poor quality of life, represents a major therapeutic challenge in Parkinson’s disease (PD). The efficacy of acupuncture for PD remains unclear, largely due to methodological flaws and lack of studies using objective outcome measures.

**Objective:**

To objectively assess the efficacy of electroacupuncture (EA) for gait disorders using body-worn sensor technology in patients with PD.

**Methods:**

In this randomized pilot study, both the patients and assessors were masked. Fifteen PD patients were randomly assigned to an experimental group (n = 10) or to a control group (n = 5). Outcomes were assessed at baseline and after completion of three weekly EA treatments. Measurements included gait analysis during single-task habitual walking (STHW), dual-task habitual walking (DTHW), single-task fast walking (STFW), dual-task fast walking (DTFW). In addition, Unified Parkinson's Disease Rating Scale (UPDRS), SF-12 health survey, short Falls Efficacy Scale-International (FES-I), and visual analog scale (VAS) for pain were utilized.

**Results:**

All gait parameters were improved in the experimental group in response to EA treatment. After adjustment by age and BMI, the improvement achieved statistical significant level for gait speed under STHW, STFW, and DTFW (9%-19%, p<0.05) as well as stride length during DTFW (9%, p = 0.037) and midswing speed during STFW (6%, p = 0.033). No significant changes were observed in the control group (p>0.110). The highest correlation between gait parameters and UPRDS scores at baseline was observed between gait speed during STFW and UPDRS II (r = -0.888, p = 0.004). The change in this gait parameter in response to active intervention was positively correlated with baseline UPDRS (r = 0.595, p = 0.057). Finally, comparison of responses to treatment between groups showed significant improvement, prominently in gait speed (effect size 0.32–1.16, *p* = 0.001).

**Conclusions:**

This study provides the objective proof of concept for potential benefits of non-pharmaceutical based EA therapy on enhancing gait in patients with PD.

**Trial Registration:**

ClinicalTrials.gov NCT02556164

## Introduction

The hypokinetic rigid gait of PD is mainly characterized by a reduction of gait speed and stride length [1–3]. These symptoms progress with time in the majority of cases, leading to festination, freezing of gait, and falls [4]. Gait disorders as other axial motor symptoms such as dysphagia, fall and postural instability tend to be treatment-resistant [5]. Dopamine treatment and deep brain stimulation (DBS) have limited efficacy and in some cases even result in worsened gait in PD [4, 6, 7]. In light of the significant limitations of conventional therapy, interest in complementary and alternative therapies for PD is growing, with acupuncture emerging as one of the most popular alternative therapies [8–10].

Despite the widespread use of acupuncture in recent years, its efficacy remains unclear, largely due to methodological flaws and lack of high quality studies using objective outcome measures [11]. An open-label clinical trial of acupuncture in PD suggested that no significant changes were noted in motor or depression scores although there was subjective improvement in some symptoms such as tremor, handwriting, difficulty walking, and sleep [10]. A double-blind randomized pilot study compared acupuncture to a control with a nonacupuncture procedure, and found no significant changes in the outcomes measured, including the Unified Parkinson’s Disease Rating Scale (UPDRS) [12]. One randomized pilot study, however, did show significant improvement in the UPDRS and 30 meter walking time [13].

Recently, objective gait and balance measurement using body-worn sensors has been employed as surrogate endpoints for demonstrating clinical efficacy of new treatments, in place of counting falls from diaries, using stop-watch measures gait speed, or relying on clinical rating scales [14]. Our previous report has shown improvement in balance following electro-acupuncture (EA) using body-worn sensors in PD patients [15]. To our knowledge, gait has not been objectively quantified by using such biomechanical assessment in any PD acupuncture study. Moreover, potential acupuncture effects on attention-related motor performances, such as dual-task walking in PD, have not been evaluated, even though dual-task gait performance is strongly associated with fall risk and predicts disability in PD [16]. Finally, it is unclear if the stage of PD affects the response to acupuncture treatment [10, 17].

In this pilot study, we addressed the methodological pitfalls of earlier studies by applying objective innovative body-worn sensor technologies for assessing potential mobility-associated outcomes of PD therapy. We compared acute changes in gait that occurred after repeated administration of two interventions: A specific real EA and a sham EA. The design of our experiments uses a control that accounts for both placebo and possibly some active components of a generalized needle-insertion-based procedure. By using a sham control, we were able to fully assess whether our specific EA intervention could lead to changes in objective gait parameters, or subjective self-reported improvements that are beyond placebo induced effects and the natural course of the disease. We demonstrated that EA is safe and effective for gait disorders in patient with PD.

## Methods

### Standard protocol approvals, registrations, and patient consents

The protocol for this trial and supporting CONSORT checklist are available as supporting information (S1 Protocol, S1 CONSORT Checklist). Participants were consecutively recruited from May 2013 to April 2014 at the movement Disorder Clinic, the University of Arizona (HL, SS). The study was approved by the University of Arizona Institutional Review Board and written informed consent was obtained from all subjects before participation. The intervention employed routine acupuncture techniques in this clinic so the study was not registered initially. The authors confirm that all ongoing and related trials for this intervention are registered in ClinicalTrials.gov with the Identifier NCT02556164. https://clinicaltrials.gov/ct2/show/NCT02556164?term=acupuncture+and+gait&rank=1

**Fig 1:**
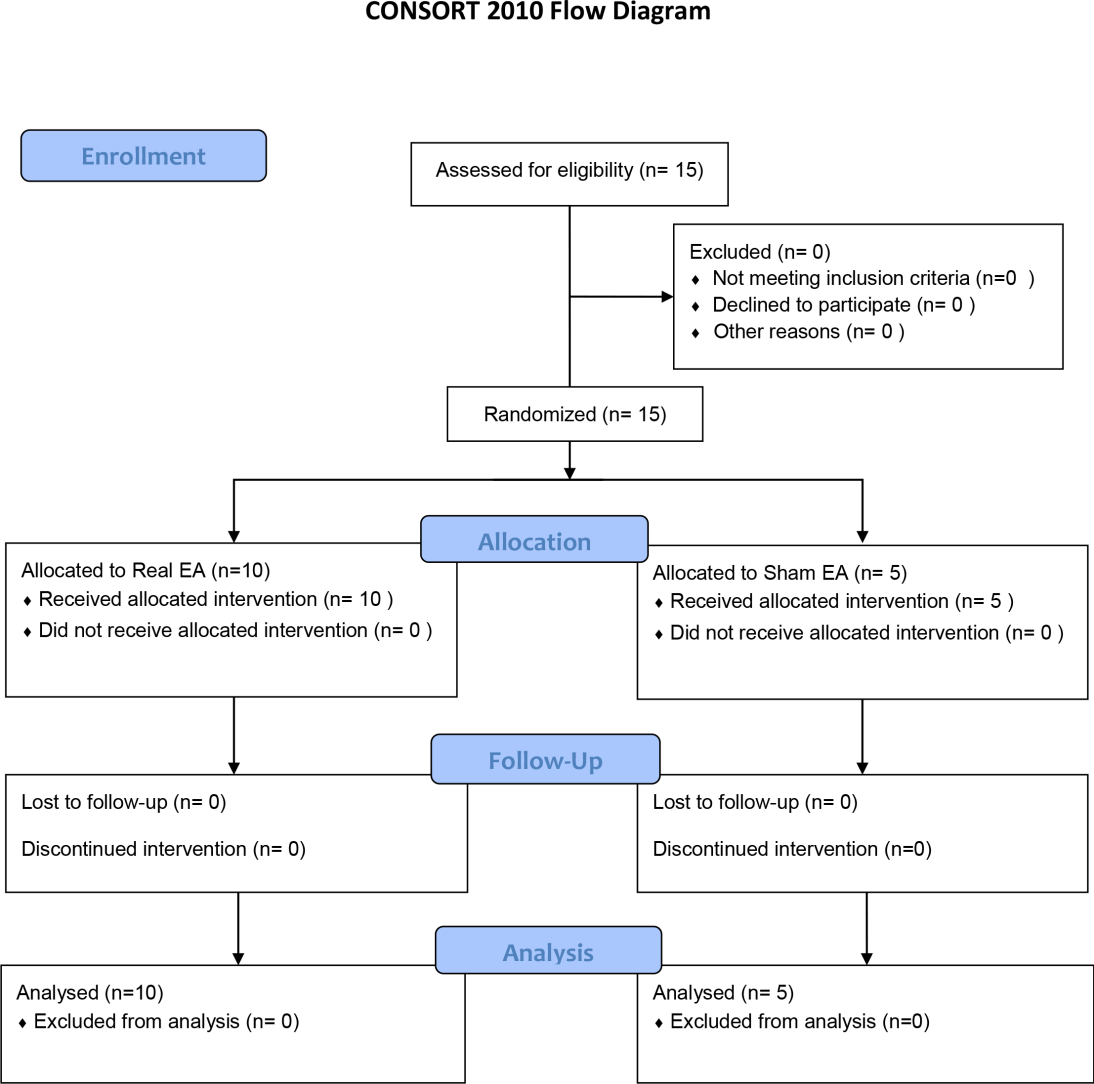
CONSORT Flow Diagram.

Participants were randomly assigned (NT) with a ratio of 2 to 1 to either the experimental (n = 10) or the control (n = 5) groups (Fig 1). Randomization was done by drawing pieces of paper from a bag. The patients in the experimental group received treatment at standard acupuncture points, and in the control group at placebo points. All patients underwent either real or sham acupuncture for 3 weeks in once-weekly, 30-minute sessions. Outcomes were assessed in an equal or greater than 12 hours defined off anti-Parkinsonian medication state at both baseline and 30 minutes after the completion of the final treatment. All assessors were masked to treatment allocation.

Inclusion Criteria for the study include: 1) Community-dwelling men or women ages 55 years or older with diagnosis of PD; 2) patients who have the ability to walk 20 meters without walking assistance; and 3) patients who are stable without anti-PD medication(s) change for at least 1 month. Calculation of a daily L-dopa equivalent daily dose (LEDD) was based on theoretical equivalence to L-dopa as follows (modified from Evans AH, et al., 2004 and Wüllner U, et al., 2010):[18, 19] LEDD = regular levodopa dose x 1 + levodopa continuous release dose x 0.75 + ([regular levodopa dose + continuous release levodopa dose x 0.75] x 0.25 if taking tolcapone or entacapone) + pramipexole dose x 67 + ropinirole dose x 20 + rotigotine x 30 + pergolide dose x 100 + bromocriptine dose x 10 + cabergoline dose x 50 + amantadine dose x 0.5 + selegiline dose x 10 + resagiline dose x 100. The PD diagnosis was made by movement disorder specialists based on the UK Brain Bank criteria [20], and supported by DaTscan (Ioflupane I 123 injection) when possible. Exclusion Criteria for the study include: 1) patients who have received previous acupuncture; 2) patients who have had DBS; 3) patients with any clinically significant medical condition, psychiatric condition, drug or alcohol abuse, or laboratory abnormality that would, in the judgment of the investigators, interfere with the ability to participate in the study; and 4) patients with non-PD related gait disorders.

### Electro-acupuncture procedure

A standardized EA regimen, consisting of scalp and body acupuncture, with a focus on improving balance and gait for PD patients was designed and administered by a single physician (HL), who had more than 5 years of experience in acupuncture and was certified by the American Board of Medical Acupuncture and the American Board of Psychiatry and Neurology. Sterile disposable, surgical stainless steel acupuncture needles (Seirin, L type, Japan), measuring 0.25 mm in diameter and 40–50 mm in length were used for acupuncture, and 3 pocket portable electric stimulators (ITO ES-130, Japan) were used for EA stimulation. Each of the electrical stimulator has 3 independent intensity channels with a pulsed current. The pulse was asymmetric biphasic square wave with pulse width 100 microseconds (μS). Real EA was performed at standard acupuncture points with depth and frequencies as shown in Table 1. “De-qi” was achieved with needle manipulation. In the treatment EA group, the current amplitude (intensity) was adjusted and recorded as number of dial between 3.5 and 4.5, just below the level that induces visible muscle contraction. All the acupuncture points were stimulated at the same time with frequency of 100 Hz or 4 Hz. The Sham acupuncture was performed for the control group with insertion of needles less than 4mm, just under the skin at non-acupuncture points (at the same areas for all subjects) at scalp, neck, shoulder, upper and lower extremities without needle manipulation. The electric stimulation in sham acupuncture was performed in a similar fashion to the real EA but with intensity equal to zero. The treating physician was responsible for recording adverse events and safety assessments.

**Table 1:** Acupuncture procedure in experimental group.

Point	Location	Insertion	Frequency (Hz)
Foot Motor Sensory Area	1 cm left or right to the midpoint of midline, extending up to 4cm posteriorly.	Transversely	100
Balance Area	Starting at occipital protuberance, 3.5-4cm to the left or right of midline, then going downwards 4cm.	Transversely	100
GV20 (Baihui)	The midpoint of the connecting line between the auricular apices when the ears are folded.	Transversely	4
GV14 (Dazhui)	On the posterior midline, in the depression inferior to the spinous process of the 7^th^ cervical vertebra (C7)	Perpendicularly	4
LI4 (Hegu)	On the dorsum of the first interosseus space of the hand, at the level of the midpoint of the shaft of the 2^nd^ metacarpal bone.	Perpendicularly	100
ST36 (Zusanli)	On the superolateral aspect of the leg, 3 Cun distal to the inferior border of the patella and one finger-breath lateral to the tibial tuberosity, in a muscle groove.	Perpendicularly	100
GB34 (Yanglingquan)	On the lateral side of leg, in a depression anterior and inferior to the head of the fibula.	Perpendicularly	100
BL40 (Weizhong)	At the midpoint of the popliteal crease.	Perpendicularly	4
SP6 (Sanyinjiao)	On the medial side of the leg, posterior to the medial margin of the tibia, 3 Cun above the prominence of the medial malleolus.	Perpendicularly	4
KI3 (Taixi)	On the posteromedial aspect of the the ankle, in the depression between the prominence of the medial malleolus and Achilles tendon.	Perpendicularly	4
LR3 (Taichong)	On the dorsum of the foot, on the first and second metatarsal bones, in the depression distal to the junction of the bases of the two bones, over the dorsalis pedis artery	Perpendicularly	100

### Measurements

Primary outcome measure was steady-state gait speed. Secondary outcome measures were other steady-state spatio-temporal gait parameters including stride length, cadence, double support, and midswing angular velocity, the UPDRS, SF-12 health survey, short Falls Efficacy Scale-International (FES-I), and visual analog scale (VAS) for pain. Participants performed four bouts of gait test (> 25 steps) during unobstructed single-task habitual walking (STHW), dual-task habitual walking (DTHW), single-task fast walking (STFW), and dual-task fast walking (DTFW). Angular velocity of shanks, thighs and trunk were measured using wearable sensor technologies (LEGSys™ [BioSensics LLC, Boston, MA]) to derive gait outcome measures following identical procedures explained in earlier studies [21, 22]. Double support was measured as the percentage of time in each stride cycle when both feet are in contact with the ground. Midswing angular velocity was measured as the shank angular velocity peak in the middle of swing phase in each cycle and averaged cross steady state cycles. The beginning of the steady-state walking was quantified as the first stride of the group of six strides with an standard deviation (SD) below the median SD of all the analyzed strides ±6% [22]. All above measurements were performed by trained examiners at baseline and after the completion of 3 weeks’ treatment. In addition, modified Hoehn and Yahr (H&Y) staging, and Mini-Mental State Examination (MMSE) were obtained at baseline.

### Statistical analysis

We used descriptive statistics to characterize demographics and performance for each group. Repeated measures analysis of covariance (ANCOVA) was used to compare the effect of the intervention on outcome parameters post intervention adjusting for age and BMI. An independent-samples t-test (or Mann–Whitney U test for non-Gaussian samples) was used for determining differences between groups except for participant’s gender, in which, a Fisher’s exact test was used. Due to small sample size, Bootstrap was also applied to Independent-samples t-test for comparison of responses to treatment between experimental and control groups. Bias-corrected and accelerated method with 1000 replications and default seed were chosen with age as strata variable. Respectively, parametric or non-parametric approach was taken for normally distributed or not normally distributed samples. To assess the normality goodness for each sample we used the Shapiro-Wilk W test. A Bivariate Correlation (Spearman’s rank) was utilized in the experimental group to assess the relationship between response to treatment (for outcomes significantly improved) and participants’ baseline characteristics such as age, H&Y staging, MMSE or UPDRS III. Cohen’s d was used for calculating effect size [23]. A *p-*values less than 0.05 (two-tailed) was considered statistically significant. All analyses were performed using SPSS Version 23.

## Results

Baseline characteristics of participants are detailed in Table 2. No difference was observed between experimental and control groups at baseline for assessed parameters. All participants completed the three intervention sessions and tolerated procedures without significant adverse effects. However, one patient in the experimental group reported transient lightheadedness at near the end of final treatment. Subjects were not able to determine whether they received real or sham acupuncture (χ^2^ = 1.875, *p* = 0.17).

**Table 2:** Baseline characteristics of the study population.

Mean (SD or percentage)	Experimental	Control	Total	*p*-value
Number (% of total)	10 (67%)	5 (33%)	15	-
Male (% of the group)	6 (60%)	2 (40%)	8 (53%)	0.46
Age (yr)	69.8 (4.5)	71.0(11.7)	70.2 (7.3)	0.78
Height (cm)	164.6 (10.1)	163.6 (13.5)	164.3 (10.9)	0.87
BMI (kg/m^2^)	27.5 (4.1)	28.1 (6.5)	27.5 (6.5)	0.85
Disease duration (yr)	6.2 (5.9)	5.2 (4.7)	5.9 (5.3)	0.75
Total LEDD	614 (381)	324 (295)	517 (380)	0.17
Dopamine agonist LEDD	54 (102)	60 (66)	56 (90)	0.72
H&Y stage	3.0 (1.0)	2.9 (0.7)	2.9 (0.9)	0.92
UPDRS I	5.3 (3.5)	3.3 (2.4)	4.6 (3.2)	0.28
UPDRS II	18 (9.7)	17 (5.7)	17.7 (8.4)	0.84
UPDRS III	35.1 (15.3)	34.2 (12.3)	34.8 (13.9)	0.91
MMSE	24.9 (6.7)	27.2 (1.1)	25.7 (5.6)	0.47

LEDD = regular levodopa dose x 1 + levodopa continuous release dose x 0.75 + ([regular levodopa dose + continuous release levodopa dose x 0.75] x 0.25 if taking tolcapone or entacapone) + pramipexole dose x 67 + ropinirole dose x 20 + rotigotine x 30 + pergolide dose x 100 + bromocriptine dose x 10 + cabergoline dose x 50 + amantadine dose x 0.5 + selegiline dose x 10 + resagiline dose x 100. H&Y = Hoehn and Yahr; UPDRS = Unified Parkinson's Disease Rating Scale; MMSE = Mini-Mental State Examination. An independent-samples t-test (or Mann–Whitney U test for non-parametric samples) was used for determining differences between groups except for participant’s gender, in which, a Fisher’s exact test was used.

### Gait analysis

**Fig 2:**
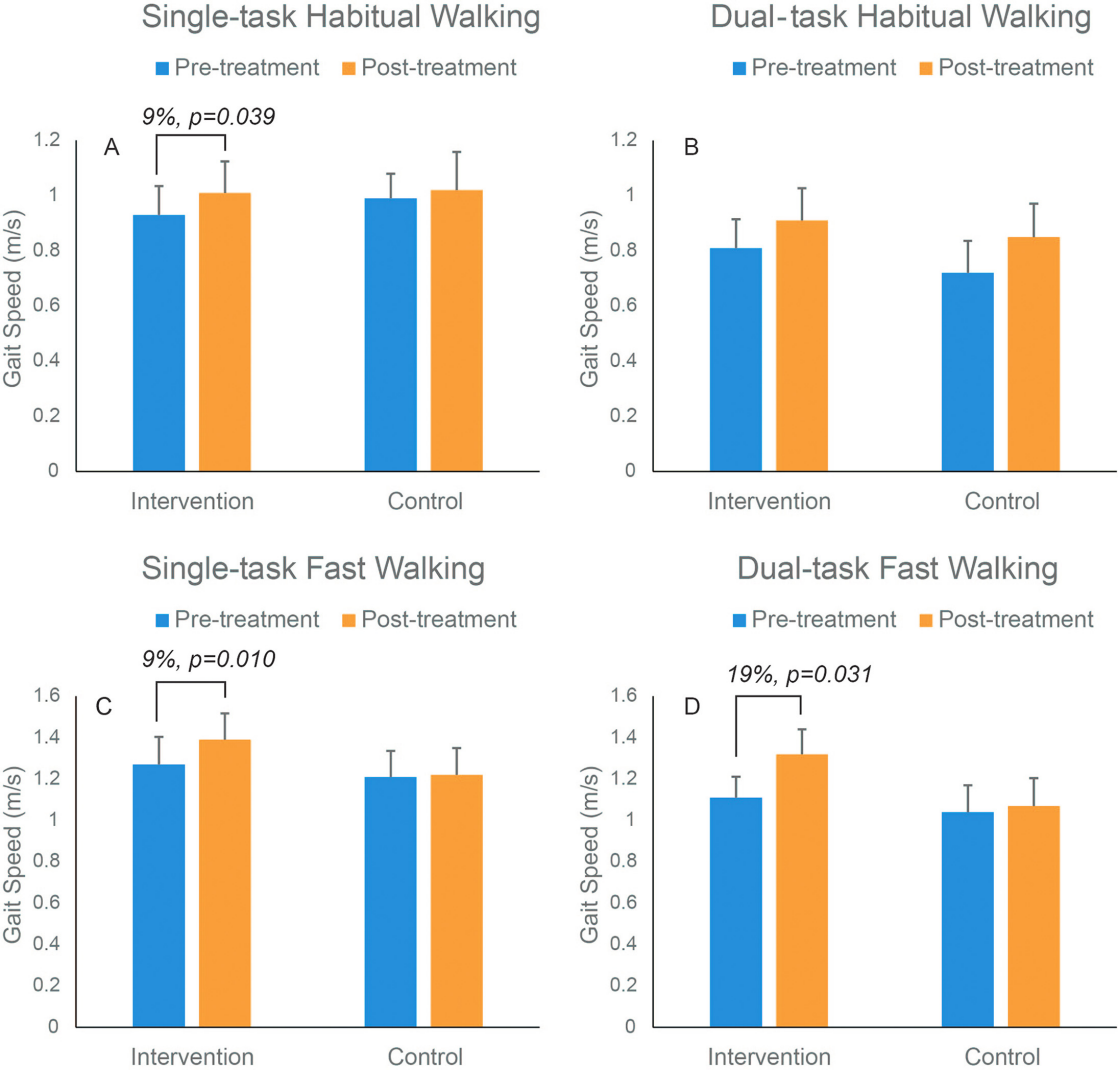
Changes in gait speed pre- and post-treatment in the intervention group (blue color) and control group (orange color) during single-task habitual, dual-task habitual, single-task fast and dual-task fast walking conditions. Mean values and standard errors are illustrated. Only the values for those parameters, which achieved statistical significant level were illustrated.

Two out of 15 participants had partial missing gait data and they were both in the experimental group. One patient refused to perform fast walking at the baseline and another patient had invalidated measurements during fast walking. These missing gait data were omitted from the statistical analysis. Table 3 shows comparison of responses to EA in gait parameters within experimental or control groups. Overall, all gait parameters were improved in the intervention group in response to EA treatment after adjustment with age and BMI. Specifically, as illustrated in Fig 2, significant improvement occurred in the experimental group compared to baseline for gait speed during STHW (9%, *p* = *0*.*039*), STFW (9%, *p* = *0*.*010*), and DTFW (19%, *p* = 0.031). Stride length was increased under all tested conditions but observed improvements were only significant for STHW (13%, *p* = 0.023) and DTHW (9%, *p =* 0.037). Similarly, a trend of improvement was noted for cadence, double support and midswing angular velocity (indicator of joint rigidity) across all conditions. But only midswing speed improvement during STFW achieved statistically significant level in our sample (6%, p = 0.033) after adjustment with age and BMI. With significantly increased cadence (12%, *p* = 0.048) during DTFW and increased midswing angular velocity during DTFW (16%, *p* = 0.029). No significant improvement was determined in any measured gait parameters in the control group compared to baseline (*p*>0.061). Finally, comparison of response to treatment between experimental and control groups showed significant improvement in most of the measured gait parameters (S1 Table), most prominently in gait speed during all walking conditions (effect size 0.32–1.16, *p* = 0.001).

**Table 3:** Comparison of responses to treatment in gait parameters within experimental or control groups.

		Experiment						Control					
Gait Parameter	Walking Condition	Pre-EA Mean (SD)	Post-EA Mean (SD)	% Change	95% CI Lower	95% CI Upper	p-value (η_p_^2^)	Pre-EA Mean (SD)	Post-EA Mean (SD)	% Change	95% CI Lower	95% CI Upper	p-value (η_p_^2^)
Speed (m/s)	STHW	0.93 (0.33)	1.01 (0.36)	9% ↑	-0.16	-0.01	.039*	0.99 (0.20)	1.02 (0.31)	3% ↑	-0.33	0.27	0.707
	DTHW	0.81 (0.33)	0.91 (0.37)	12% ↑	-0.25	0.06	0.182	0.72 (0.26)	0.85 (0.27)	18% ↑	-0.51	0.25	0.285
	STFW	1.27 (0.42)	1.39 (0.40)	9% ↑	-0.24	-0.01	.010*	1.21 (0.28)	1.22 (0.29)	1% ↑	-0.16	0.13	0.715
	DTFW	1.11(0.32)	1.32 (0.38)	19% ↑	-0.4	-0.03	.031*	1.04(0.29)	1.07 (0.30)	3% ↑	-0.12	0.08	0.411
Stride (m)	STHW	1.08 (0.30)	1.13(0.27)	13% ↑	-0.1	-0.01	.023*	1.17 (0.22)	1.21 (0.23)	3% ↑	-0.2	0.13	0.424
	DTHW	1.04 (0.34)	1.09 (0.29)	5% ↑	-0.14	0.04	0.247	1.01 (0.19)	1.10 (0.26)	9% ↑	-0.31	0.13	0.224
	STFW	1.21 (0.28)	1.26 (0.30)	4%↑	-0.12	0.01	0.058	1.26 (0.25)	1.27 (0.26)	1% ↑	-0.12	0.1	0.768
	DTFW	1.17 (0.19)	1.27 (0.25)	9% ↑	-0.2	-0.01	.037*	1.19 (0.26)	1.20 (0.30)	.2% ↓	-0.04	0.03	0.555
Cadence (steps/min)	STHW	50.2 (6.92)	52.5 (8.61)	4% ↑	-5.76	1.12	0.155	50.8 (4.64)	49.7 (8.64)	2% ↓	-14.9	17.1	0.799
	DTHW	45.9 (8.12)	48.5(10.12)	6% ↑	-8.48	3.3	0.333	42.4 (9.59)	45.6 (7.4)	8% ↑	-14.2	7.7	0.334
	STFW	61.3 (7.96)	65.4 (8.05)	7% ↑	-9.39	1.26	0.107	57.2 (5.3)	57.3 (6.5)	0% ↑	-2.1	1.9	0.878
	DTFW	55.7 (10.6)	61.1(7.83)	10% ↑	-10.8	0.04	0.051	51.6 (6.8)	53.4 (6.4)	3% ↑	-8.1	4.7	0.369
Double Support (%)	STHW	27.7 (7.6)	25.1 (7.5)	10%↓	-1.3	6.5	0.164	22.7 (2.2)	23.3 (4.9)	3% ↑	-10.3	8.9	0.796
	DTHW	32.2 (13.1)	27.7 (8.7)	14% ↓	-1.3	10.1	0.111	29.0 (7.5)	26.0 (4.6)	10% ↓	-11.3	17.2	0.469
	STFW	19.5 (4.7)	16.9 (5.4)	13% ↓	-2	7.3	0.203	20.1 (5.5)	17.9 (2.3)	11% ↓	-.6.2	10.5	0.382
	DTFW	21.8 (4.8)	19.6 (6.0)	10% ↓	-1.5	6.1	0.186	24.3 (5.4)	19.9 (2.6)	18% ↓	-.8.0	16.7	0.269
Midswing (degree/s)	STHW	273 (93.8)	287 (95.9)	6% ↑	-31.4	2.6	0.085	292 (52.3)	304 (80.3)	4% ↑	-111	88	0.673
	DTHW	250 (89.4)	259 (92.6)	4% ↑	-43.8	26.2	0.57	230 (69.6)	260 (56.4)	13% ↑	-119	60	0.288
	STFW	343 (111)	365 (87.9)	6% ↑	-40.4	-2.6	.033*	337 (62.8)	354 (65.7)	5% ↑	-60	25	0.224
	DTFW	305 (88.1)	345 (81.9)	13% ↑	-81.4	1.2	0.055	297 (60.4)	318 (56.4)	7% ↑	-55	12	0.11

STHW = single-task habitual walking, DTHW = dual-task habitual walking, STFW = single-task fast walking, DTFW = dual-task fast walking. CI = Confidence Intervals.

* indicates a significant difference (ANCOVA, *p*<0.05).

↓ indicates a reduction and,

↑ indicates an increase in each parameter following the treatment.

### UPDRS and subjective reports

As demonstrated in one of our previous reports [15], there was an overall improvement (S2 Table) within experimental group in mentation, behavior, and mood (UPDRS I 49%, *p*<0.01), in the activity of daily living (UPDRS II, 46%, *p*<0.0001) and motor examination (UPDRS III, 40%, *p*<0.01), whereas, these changes were small and non-significant in the controls (*p*>0.21). Although changes were not significant, short FES-I and VAS scores were reduced by 15% (*p* = 0.41) and 44% (*p* = 0.26), respectively, following treatment in the experimental group. Comparison of response to treatment between experimental and control groups showed significant improvement (See S1 Table in UPDRS I (effect size 1.82, *p* = 0.005), UPDRS II (effect size 1.49, *p* = 0.02) and UPDRS III (effect size 3.30, *p*<0.001).

### Correlations

**Fig 3:**
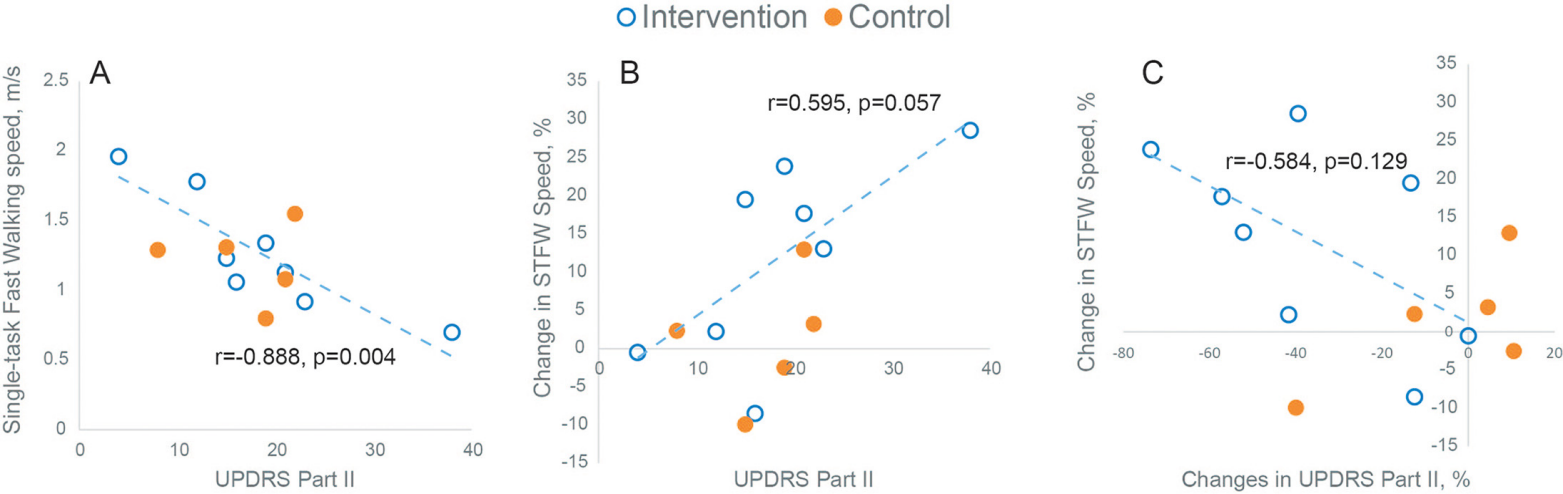
(A) Association between baseline gait speed during Single Task Fast Walking (STFW) condition and baseline UPDRS Part II. The negative correlation suggests that those with poor UPDRS score walk slower in the intervention (real EA) group; (B) Association between changes in magnitude of gait speed during STFW in response to EA treatment and baseline UPDRS Part II. The positive correlation suggests that those with poor baseline UPDRS score may benefit more from EA treatment. (C) Association between changes in magnitude of gait speed during STFW and changes in magnitude of UPDRS. Negative correlation suggests that enhancement in gait speed in response to EA treatment is associated with enhancement in UPDRS score.

Negative correlation was observed in the intervention (real EA) group between gait speed and UPDRS scores at both baseline and follow-up with the strongest correlation between UPDRS part II and gait speed during single task fast walking condition (r = -0.888, p = 0.004, Fig 3A). A positive correlation was observed between changes in gait speed in response to EA intervention and baseline UPDRS score with the strongest correlation during single task fast walking condition (r = 0.595, p = 0.057, Fig 3B), indicating that patients with more impairment in activity of daily living may benefit more from EA treatment. Results also suggest that enhancement in gait speed in response to EA treatment, specifically during STFW, is associated with enhancement in UPDRS score, in particular for UPDRS Part II (r = -0.584, p = 0.129). However, this association didn’t achieve statistical significant level, probably due to small sample size. A negative correlation between gait speed improvement during STHW and age (r = -0.585, p = 0.049) was observed in the experimental group. No other significant correlation was observed between magnitude of gait improvement in response of EA treatment and baseline (H&Y) staging, or MMSE (*p*>0.153).

## Discussion

To the best of our knowledge this is the first participants- and assessors- masked randomized control study that investigated potential benefits of EA in enhancing spatio-temporal parameters of gait in PD patients as objectively measured using wearable sensor technology. By comparing a group of patients who received real EA to those in the sham control, we found that patients in the experimental group significantly improved in gait performance after completion of the 3-week intervention. Placebo effects have been known to be caused by increased patient-physician contact or patient belief in the effectiveness of treatment, and have been postulated to relate to insufficient masking. To address these issues, we utilized sham acupuncture and masking of the patients as well as the assessors. The patients in both groups are naïve to acupuncture prior to this study. All patients received identical contact from the research team, standardized treatment and objective gait measurements. By adhering to these measures, we attempted to exclude placebo effects in the patients and contamination by the assessors. Therefore, these beneficial effects of the selected acupuncture regimen found within experimental group (comparison of pre and post EA) and between experimental and control groups can be regarded as specific to the treatment.

The results indicated an overall beneficial impact on different spatio-temporal parameters of gait such as gait speed and stride length as well as gait parameters related to dynamic postural control such as increased midswing angular velocity and decreased double support. The later results suggest an enhanced efficiency of balance control during gait [24] and are in agreement with our previous report that EA has beneficial effects on balance [15]. Moreover, evidence suggested that patients with PD usually develop cognitive decline and other non-motor problems that impair their ability to deal with multiple simultaneous tasks and revealed the insufficiency of compensatory motor mechanisms [16, 25]. Accordingly, our data showed that gait performance improved during single-task and more importantly during dual-task walking. Finally the correlation analysis suggests that patients with more impairment in activity of daily living may benefit more from EA treatment. Our clinical assessment also revealed improvement in motor and non-motor signs and symptoms, as well as subjective report of improved activity of daily living by using UPDRS and questionnaires.

Some of key well-known caveats of the clinical scales such as the UPDRS are its rater and time-of-assessment dependency. The use of wearable sensor technology could address these shortcomings by objectively measuring motor performance changes in response to treatment. The key advantages of wearable technologies are their ability to capture gait and balance characteristics irrespective of environment of test (in clinic or home) outside of gait laboratory environment with less time consumption (usually less than 20 minutes). Our results suggested that motor performance measured using wearable technologies are sensitive enough to track treatment responses to alternative therapies. Using objective modalities instead of subjective tools to evaluate treatment response to alternative therapies are particularly important when exclusion of placebo effect is difficult. All the gait measurements using body worn sensors in this study were performed in a clinical setting within a short duration of time and were well tolerated by PD patients.

Acupuncture, an alternative medicine methodology that originated in ancient China, treats patients using various techniques, including inserting small, thin needles at specific points in the body. EA, as the name implies, combines classical acupuncture and low electric current running through the needles, in order to enhance a treatment. Compared with manual manipulation in classical acupuncture, electric manipulation in EA can be standardized by selecting the type, frequency and intensity of stimulation. In the study reported here, a novel standardized EA regimen, with a focus on improving gait and balance, has been developed based on the principles of Traditional Chinese Medicine (TCM), pathophysiology of gait in PD, previous studies [13, 26–29], and our clinical experience.

The exact mechanisms behind the beneficial effects of EA in gait disorder are unclear. However, evidence from PD animal models [30, 31] suggested that acupuncture may enhance neurotransmission, trophic factor release, reduction of apoptotic and oxidative damages, improvement of synaptic plasticity and modulation of activity in the basal ganglia circuits. Moreover, human studies with functional magnetic resonance imaging (fMRI) have shown that acupuncture can influence brain networks by activating the precentral gyrus and prefrontal cortex in PD [31, 32].

We believe the beneficial effects of EA on gait observed in this study are, at least partially, due to improved motor (e.g. rigidity) and mental function after EA. In addition, EA could also improve gait through directly modulating brain networks such as locomotor and balance networks, as well as the cortex for higher-level gait disorder. Additional investigation with modern technology such as fMRI and diffusion tensor imaging could provide further insights into the mechanisms involved.

While fewer acupuncture points have been used in previously published PD related studies, in the present study we selected a total of 20 points (9 paired points and 2 single points). The procedure was well tolerated by all subjects with no serious adverse effects. No participant discontinued the study after initial consent was given. Our zero dropout rate and low adverse effects highlight the safety and feasibility of EA in PD.

There are limitations in this study: First, this pilot study has a small sample size. Although there was a trend for superior efficacy of real EA, a clinical trial with larger sample with objective randomization and further control (e.g., different freezing gait subtypes) is needed to confirm the benefits of the described intervention. Second, in this study, the number of treatment sessions was based on the combination of expected minimum treatments required in our practice and a realistic assessment of the number of sessions that a participant could attend without posing an undue hardship. It is possible that different regimens such as more frequent and/or longer treatment periods might provide more robust effect of benefits. In addition, the decision to use pre-set protocols was made in an attempt to standardize the treatment. However, the potential drawback of this approach is a possible loss in the efficacy of the acupuncture treatment due to a lack of individualized, comprehensive treatments in the way that acupuncture is traditionally performed. Third, the duration of improvements observed in gait performance was rather short after the EA treatment. Our ongoing research is extending EA therapy to ascertain whether or not there will be more sustained long term effects. Finally, randomization was done by drawing pieces of paper from a bag, which may not be fully objective. A more objective randomization order (e.g., ordering of the subjects in alphabetical order, and assigning them to treatment and control, respectively, by a random number generator with seed option) is recommended for future studies.

## Conclusions

This study provides an objective proof of concept for potential benefits of non-pharmaceutical-based EA therapy on enhancing gait in patients with PD. EA used in this study is simple, safe, and effective as an alternative and complementary treatment for gait disorders in PD.

## Supporting Information

S1 CONSORT Checklist (DOC)

S1 Clinical Trial ID (PDF)

S1 Protocol (DOCX)

S1 Supporting Data (XLSX)

S1 Table Comparison of responses to treatment between experimental and control groups. (DOCX)

S2 Table Comparison of responses to treatment within experimental and control groups in subjective reports and UPDRS. (DOCX)
