# Supplementary material for: A Pilot Clinical Trial to Objectively Assess the Efficacy of Electroacupuncture on Gait in Patients with Parkinson's Disease Using Body Worn Sensors
Source: PLoS One. 2016 May 26;11(5):e0155613. doi: 10.1371/journal.pone.0155613 (PMC4882016; doi:10.1371/journal.pone.0155613)
Supplement: S1 Clinical Trial ID — (PDF) [file pone.0155613.s002.pdf]

ClinicalTrials.gov PRS
Protocol Registration and Results System

ClinicalTrials.gov PRS DRAFT Receipt (Working Version)
Last Update: 09/21/2015 10:39

ClinicalTrials.gov ID: NCT02556164

Study Identification

Unique Protocol ID: UArizona

Brief Title: Electro-acupuncture for Gait and Balance in Parkinson's Disease

Official Title: Objective Assessment of Electro-acupuncture Efficacy for Gait and Balance in Patients With Parkinson's Disease

Secondary IDs:

Study Status

Record Verification: September 2015

Overall Status: Active, not recruiting

Study Start: May 2013

Primary Completion: May 2016 [Anticipated]

Study Completion: May 2016 [Anticipated]

Sponsor/Collaborators

Sponsor: University of Arizona

Responsible Party: Principal Investigator
Investigator: Hong Lei [hlei]
Official Title: Assistant Professor of Clinical Neurology
Affiliation: University of Arizona

Collaborators:

Oversight

FDA Regulated?: No

IND/IDE Protocol?: No

Review Board: Approval Status: Approved
Approval Number: 12-0616
Board Name: Institutional Review Board
Board Affiliation: University of Arizona
Phone: (520) 626-6721
Email: VPR-IRB@email.arizona.edu

Data Monitoring?: Yes

Oversight Authorities: United States: Institutional Review Board

Study Description

Brief Summary: Gait and balance disorders, key contributors to fall and poor quality of life, represent a major therapeutic challenge in Parkinson's disease (PD). Despite the widespread use of acupuncture in recent years in PD, its efficacy remains unclear, largely due to methodological flaws and lack of high quality studies using objective outcome measures. In a patient and assessor-blind pilot study, investigators objectively assess the efficacy of electroacupuncture (EA) for gait and balance disorders using body-worn sensor technology in patients with PD.

Detailed Description: In this study, investigators employee objective innovative body-worn sensor technologies to assess potential mobility-associated outcomes of PD. Investigators compare acute changes in gait and balance that occurred after repeated administration of two interventions: A specific real EA and a sham EA. The design of the experiments uses a control that accounts for both placebo and possible some active components of a generalized needle-insertion-based procedure. By using a sham control, the investigators were able to more fully test whether the specific EA intervention could lead to changes in objective gait and balance parameters, or subjective self-reported improvements that are beyond placebo induced effects and the natural course of the disease.

Conditions

Conditions: Parkinson's Disease

Keywords: Electroacupuncture
Body Worn Sensor
Gait
Balance

Study Design

Study Type: Interventional

Primary Purpose: Treatment

Study Phase: N/A

Intervention Model: Parallel Assignment

Number of Arms: 2

Masking: Double Blind (Subject, Outcomes Assessor)

Allocation: Randomized

Endpoint Classification: Safety/Efficacy Study

Enrollment: 30 [Anticipated]

Arms and Interventions

Arms	Assigned Interventions
Experimental: Real EA<br>Real EA as intervention is performed at the selected standard acupuncture points and “De-qi” is achieved with needle manipulation before electric stimulation is delivered.	Procedure/Surgery: Electroacupuncture<br>Acupuncture is an alternative medicine methodology that treats patient by various techniques including inserting small, thin needles at specific points of body. Electroacupuncture

Arms	Assigned Interventions
	(EA), like the name implies, combines classical acupuncture and low electric current running through the needles, which are often used to enhance a treatment.<br>Body-worn sensor technology<br>Three-dimensional acceleration and angular velocity of shanks, thighs and the trunk were measured using wearable sensors each included a triaxial accelerometer and a triaxial gyroscope (LEGSys™ and BalanSens™ - BioSensics, Boston, MA)
Sham Comparator: Sham EA<br>Sham EA as intervention is performed for the control group at non-acupuncture points without needle manipulation. The electric stimulation in sham acupuncture was performed in a similar fashion to the real EA.	Procedure/Surgery: Electroacupuncture<br>Acupuncture is an alternative medicine methodology that treats patient by various techniques including inserting small, thin needles at specific points of body. Electroacupuncture (EA), like the name implies, combines classical acupuncture and low electric current running through the needles, which are often used to enhance a treatment.<br>Body-worn sensor technology<br>Three-dimensional acceleration and angular velocity of shanks, thighs and the trunk were measured using wearable sensors each included a triaxial accelerometer and a triaxial gyroscope (LEGSys™ and BalanSens™ - BioSensics, Boston, MA)

NOTE : No interventions have been included in Arm Description for 'Real EA'

NOTE : No interventions have been included in Arm Description for 'Sham EA'

NOTE : Intervention 'Electroacupuncture' has not been included in any Arm/Group Descriptions.

NOTE : Intervention 'Body-worn sensor technology' has not been included in any Arm/Group Descriptions.

Outcome Measures

Primary Outcome Measure:

1. Change of gait speed by objective measurement
   [Time Frame: 3 weeks] [Safety Issue: No]
   Participant performs two tests of normal gait (> 25 steps) under single task and dual task conditions. Gait speed (m/s) is assessed using body-worn sensor technology.

Secondary Outcome Measure:

2. Change of postural balance (COG) by objective measurement
   [Time Frame: 3 weeks] [Safety Issue: No]
   Each participant performs 30-second trials of balance assessment with eyes open or closed. Center of gravity (COG, cm) is assessed using body-worn sensor technology.

3. Change of stride length by objective measurement
   [Time Frame: 3 weeks] [Safety Issue: No]
   Participant performs two tests of normal gait (> 25 steps) under single task and dual task conditions. Stride length (m) is assessed using body-worn sensor technology.

4. Change of postural balance (Ankle/hip sway) by objective measurement
   [Time Frame: 3 weeks] [Safety Issue: No]
   Each participant performs 30-second trials of balance assessment with eyes open or closed. Ankle sway (deg2), Hip sway (deg2), Ankle/hip sway is assessed using body-worn sensor technology.

5. Change of Unified Parkinson's Disease Rating Scale
   [Time Frame: 3 weeks] [Safety Issue: No]
   Unified Parkinson's disease rating scale (UPDRS) is utilized. Parts I: to assess mentation, behavior and mood (0-16); Part II: to assess activities of daily living (0-52); Part III to motor assessment (0-104)

6. Change of SF-12 health survey
   [Time Frame: 3 weeks] [Safety Issue: No]
   SF-12 health survey (MCS, PCS)

7. Change of Short Falls Efficacy Scale-International
   [Time Frame: 3 weeks] [Safety Issue: No]
   (Short FES-I, 7-28)

8. Change of the visual analog scale
   [Time Frame: 3 weeks] [Safety Issue: No]
   the visual analog scale (VAS, 0-10) for pain,

9. Potential adverse events related to acupuncture
   [Time Frame: Baseline, 1 week, 2 week and 3 weeks.] [Safety Issue: Yes]
   measured by Adverse Events Report Related to Acupuncture

Eligibility

Minimum Age: 55 Years

Maximum Age:

Gender: Both

Accepts Healthy Volunteers?: No

Criteria: Inclusion Criteria:

1. Community-dwelling men or women ages 55 years or older with diagnosis of PD;
2. patients who have the ability to walk 20meters without walking assistance; and
3. patients who are stable without anti-PD medication(s) change for at least 1 month. The PD diagnosis was made by movement disorder specialists based on the UK Brain Bank criteria and supported by DaTscan (Ioflupane I 123 injection) when possible.

Exclusion Criteria:

1. patients who have received previous acupuncture;
2. patients who have had DBS;
3. patients with any clinically significant medical condition, psychiatric condition, drug or alcohol abuse, or laboratory abnormality that would, in the judgment of the investigators, interfere with the ability to participate in the study; and
4. patients with non-PD related gait disorders.

Contacts/Locations

Study Officials: Bijan Najafi, PhD
Study Principal Investigator
University of Arizona

Locations:

References

Citations:

Links:
