# Supplementary material for: A Pilot Clinical Trial to Objectively Assess the Efficacy of Electroacupuncture on Gait in Patients with Parkinson's Disease Using Body Worn Sensors
Source: PLoS One. 2016 May 26;11(5):e0155613. doi: 10.1371/journal.pone.0155613 (PMC4882016; doi:10.1371/journal.pone.0155613)
Supplement: S1 Protocol — (DOCX) [file pone.0155613.s003.docx]

Project Title: Assessing Gait And Balance Using Body Worn Sensors: Does A Successful Clinical Intervention Also Improve Gait And Balance?

Background

Gait and balance disorders are frequently found in older adults and patient populations [1-4]. They are associated with increased morbidity and mortality, as well as reduced level of function and limited quality of life [1-4]. Changes in balance and gait are usually multifactorial in origin and require a comprehensive assessment to determine contributing factors and targeted interventions [5]. In older adults alterations in gait and balance should not be viewed as merely an inevitable consequence of aging but are frequently related to underlying medical conditions [3, 5]. Gait changes such as diminished stride length, increased double limb support and stepping variability are common in patients with a variety of diseases such as Parkinson's disease (PD), diabetic neuropathy, knee and hip osteoarthritis, or hemiplegia/hemiparesis [5, 6].  Pain caused by osteoarthritis or intermittent claudication may also lead to gait and balance alterations.

Early identification of gait and balance disorders and appropriate intervention may prevent dysfunction and loss of independence. Successful intervention requires a detailed analysis of clinically relevant biomechanical parameters related to motor control during ambulation. Thus far, quantification of such parameters has been performed by optical motion measurement systems, force plates, or specific walkways for gait analysis. These assessments, however, are often not feasible during clinical practice, as they are time consuming, cost intensive, and require extensive laboratory equipment. Also, by the short walking distance offered by laboratory based-technology, it is difficult to measure the variability of gait which is important for maintaining balance and responding to different walking challenges [7].

Human body motion is traditionally captured using standard optic, magnetic or sonic technologies [8]. However, in recent years, body-wearable sensor technology based on electro-mechanical sensors (MEMS) has provided a new avenue for accurately detecting and monitoring body motion and physical activity of an individual under free conditions [8-10]. Unlike laboratory-based instruments, which need a dedicated controlled space, the wearable sensors can be used just about anywhere [8]. These are highly portable and do not require stationary units such as a transmitter, receiver or cameras. In addition, these sensors are much cheaper than sonic, magnetic and optical motion capture devices [8]. They are easy to set up and use, and do not require highly skilled operators. In particular, combination of multiple accelerometers, angular rate sensors (gyroscopes), and magnetometer show a promising design for a hybrid kinematic sensor module for measuring the 3D kinematics of different body segments [11]. These sensors incorporated with a high speed data acquisition system enable measuring and recording of 3D body segment motion with sample frequency up to several hundred hertz with lower cost than camera based systems.

Our team has designed and validated several algorithms based on body worn sensors for various clinical applications. These areas include studying the spatio-temporal parameters of gait [8, 10, 12, 13], joint and segment angles (kinematics) [11, 14-16], monitoring spontaneous daily physical activity [17-24], and evaluating the fear of falling [25], and risk of falling [26]. One of the main advantages of body-worn sensors compared to laboratory-based measuring systems is that they are ambulatory and can be used in free conditions continuously over long periods of time [10].

Assessing gait and balance using wearable sensors

The aim of the present study is to implement two validated technologies based on wearable sensors named LEGSys [10, 12, 7] and BalanSens [11] for assessing respectively spatio-temporal parameters of gait and postural control in a clinical setting together with other clinically routine assessment (Figure 1). We may utilize some ancillary non-invasive tools such as a heat sensitive thermal camera, pressure sensitive foot insoles and a digital camera to aid in collecting additional data as described in later sections. Our specific aims are twofold, first we aim to explore whether and how a disorder, which may impact motor function such as diabetes, Parkinson, Stroke, etc. may impact gait and balance. Second, we will explore whether a routinely used clinical intervention can enhance gait and balance parameters.

The LEGSys device uses five sensor modules, respectively attached to right and left anterior shins, right and left anterior thighs, and posteriorly to the lower back. Each sensor measures the angular velocity of the segment around the medio-lateral axis (flexion-extension). The method for calculating spatio-temporal parameters of gait and balance has been described in detail in previous publications [8, 12, 27].


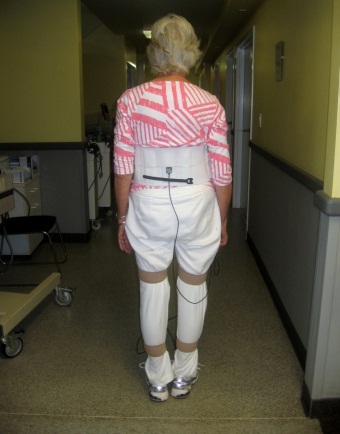

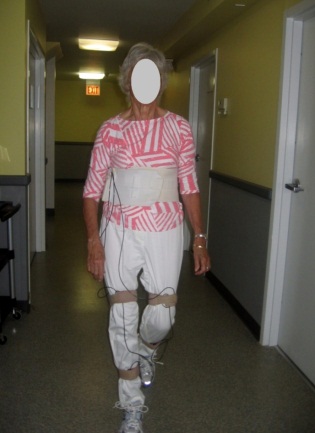


Figure 1. Wearable sensor technology used in the planned study to measure gait (left picture) and postural control (right picture).

BalanSens is based on widely-available kinematic sensors (i.e. accelerometer, gyroscope and magnetometer). The system measures ankle and hip motion in three dimensions (3D). We have also integrated the resulting data into a two-link biomechanical model of the human body for estimating the 2D sway of the center of mass (COM) in anterior-posterior (AP) and medial-lateral (ML) directions. To evaluate the best postural strategy for maintaining balance, a reciprocal compensatory index (RCI) was defined which quantifies how the movement around hip could compensate for the movement around ankle for reducing the variation of COM [11]. RCI values near zero represent a good postural control strategy (i.e. negative correlation between hip and ankle movements), RCI values more than one represent inappropriate postural control strategy (i.e. positive correlation between hip and ankle movements leading to increase the variation of COM and consequently fall accident), and RCI values near to one indicate there is no correlation between the movement of ankle and hip joints [11].

In addition to the body worn sensor systems, we will use insoles for measuring plantar pressure [Tekscan, Boston, MA] which might be related to specific diseases such as diabetes or neuropathy. Furthermore we will collect a set of relevant clinical variables including the Short Falls Efficacy Scale-International [28], Short Form Health Survey (SF-12) [29], the 5-chair rise test [30], the Cervical Spine Baseline and Cervical Spine Followup Survey, and the Lumbar Spine Baseline and Lumbar Spine Followup Questionnaires. These surveys will be conducted initially by written administration. However, we eventually will conduct them utilizing an electronic system such as Dynamic Clinical Systems interfaced with EPIC as part of standard care.

*Sample size estimation and power analysis:*

We also calculated the sample size based on our pilot study [31] using the same balance training paradigm. The results demonstrated that ankle reaching task is beneficial to improve balance by reducing the area of COM sway as well as improving the reciprocal coordination between ankle and hip movements. According to our results, we must recruit 15 patients to demonstrate a significant improvement after the proposed balance training program by assuming a power of 80% and alpha equaling 0.05. We will recruit 10 additional subjects (five per group) to compensate for potential drop out samples.

*Analysis plan:*

P-values of 0.05 or less will be considered statistically significant based on a two-sided test unless otherwise noted. All variables will be tabulated descriptively at each scheduled time point. For each continuous variable, the analyses will include the mean, standard deviation, minimum and maximum. For each categorical variable, the summary will include frequencies and percentages. Each variable will be analyzed using all available data. Baseline variables will be summarized using descriptive statistics.

To determine whether gait, balance, and physical activity variables change due to intervention (for instance, due to the balance training as described above), within-subject analyses of variance (ANOVA’s) will be employed. We assume that a successful intervention is also helpful to improve subjects gait, balance, and physical activity. Then sensor-derived movement variables should change substantially from baseline to the end of the intervention. For example, the range of motion of COM and RCI values should be reduced and gait velocity should be increased. Next, for all continuous variables, pretreatment scores will be regressed on post-treatment scores to form residualized change scores (e.g. VPT, monofilament test, pain intensity, A1C, etc). Correlations will then be generated among these scores to determine whether changes in sensor-derived movement factors are related appropriately with increases in (clinically assessed) functional capacities and self-report measures of activities, mood, socializing, etc. ANOVA test will be used to examine significant difference between intervention and sham group (if there is a sham group) or between patients and control group for age and BMI. Additional analyses will examine the degree to which early-treatment changes in cognitive variables and functional capacity variables (representing functional restoration processes) predict late-treatment changes in moving ability post intervention. Such analyses will also allow to elucidate the relative contributions fear of falling (assessed using FES score); and/or and range of motion (assessed using body worn sensors) made to bringing about clinically meaningful changes in gait balance control. Moderator effects will also be examined for exploratory purposes. Using gender as an example, mixed design ANOVA’s will be conducted to determine whether gait and balance factors change over the course of treatment at different rates or with different patterns for men and women. Additional analyses will test whether changes in gait and balance variables predict changes in clinically important outcome factors (e.g., fear of falling (FES), quality of life (SF12), adverse events, etc) depending on gender.

*References:*

Nevitt MC, Cummings SR, Kidd S, Black D. Risk factors for recurrent nonsyncopal falls. A prospective study. JAMA. 1989;261(18):2663-2668.

Rubenstein LZ, Josephson KR. Falls and their prevention in elderly people: what does the evidence show? Med Clin North Am. 2006;90(5): 807-824.

Sudarsky L. Gait disorders: prevalence, morbidity, and etiology. Adv Neurol. 2001;87:111-117.

Tinetti ME, Speechley M, Ginter SF. Risk factors for falls among elderly persons living in the community. N Engl J Med. 1988;319(26):1701-1707.

Salzman, B. (2010). "Gait and balance disorders in older adults." Am Fam Physician 82(1): 61-68

Alexander, N. B. (1996). "Gait disorders in older adults." J Am Geriatr Soc 44(4): 434-451.

Najafi, B., T. Khan, et al. (2011). "Laboratory in a box: wearable sensors and its advantages for gait analysis." Conf Proc IEEE Eng Med Biol Soc 2011: 6507-6510

Aminian, K. and B. Najafi, Capturing human motion using body-fixed sensors: Outdoor measurement and clinical applications. Computer Animation and Virtual Worlds, 2004. 15(2): p. 79-94.

Zijlstra, W. and K. Aminian, Mobility assessment in older people: new possibilities and challenges. Eur J Ageing 2007. 4: p. 3-12.

Najafi, B., et al., Does walking strategy in older people change as a function of walking distance? Gait Posture, 2009. 29(2): p. 261-266.

Najafi, B., et al., Assessing postural control and postural control strategy in diabetes patients using innovative and wearable technology. J Diabetes Sci Technol, 2010. 4(4): p. 780-91.

Aminian, K., et al., Spatio-temporal parameters of gait measured by an ambulatory system using miniature gyroscopes. Journal of Biomechanics, 2002. 35(5): p. 689-699.

Moe-Nilssen, R. and J.L. Helbostad, Estimation of gait cycle characteristics by trunk accelerometry. J Biomech, 2004. 37(1): p. 121-6.

Dejnabadi, H., et al., Estimation and visualization of sagittal kinematics of lower limbs orientation using body-fixed sensors. Ieee Transactions on Biomedical Engineering, 2006. 53(7): p. 1385-1393.

Dejnabadi, H., B.M. Jolles, and K. Aminian, A new approach to accurate measurement of uniaxial joint angles based on a combination of accelerometers and gyroscopes. Ieee Transactions on Biomedical Engineering, 2005.

Favre, J., et al., A new ambulatory system for comparative evaluation of the three-dimensional knee kinematics, applied to anterior cruciate ligament injuries. Knee Surgery Sports Traumatology Arthroscopy, 2006. 14(7): p. 592-604.

Najafi, B., Physical Activity Monitoring and Risk of Falling Evaluation in Elderly People, Ph.D. dissertation, in Electrical Engineering Department2003, Ecole Polytechnique Federale de Lausanne (EPFL): Lausanne. p. 1-178.

Salarian, A., et al., Ambulatory monitoring of physical activities in patients with Parkinson's disease. IEEE Trans Biomed Eng, 2007. 54(12): p. 2296-9.

Paraschiv-Ionescu, A., et al., Ambulatory system for the quantitative and qualitative analysis of gait and posture in chronic pain patients treated with spinal cord stimulation. Gait & Posture, 2004. 20(2): p. 113-125.

Paraschiv-Ionescu, A., et al., Nonlinear analysis of human physical activity patterns in health and disease. Phys Rev E Stat Nonlin Soft Matter Phys, 2008. 77(2 Pt 1): p. 021913.

Buchser, E., et al., Improved physical activity in patients treated for chronic pain by spinal cord stimulation. Neuromodulation, 2005. 8(1): p. 40-48.

Najafi, B., et al., Ambulatory system for human motion analysis using a kinematic sensor: Monitoring of daily physical activity in the elderly. Ieee Transactions on Biomedical Engineering, 2003. 50(6): p. 711-723.

Aminian, K., et al., Motion analysis in clinical practice using ambulatory accelerometry, in Modelling and Motion Capture Techniques for Virtual Environments1998. p. 1-11.

Najafi, B., R.T. Crews, and J.S. Wrobel, The Importance of Time Spent Standing for those at Risk of Diabetic Foot Ulceration. Diabetes Care, 2010. 33(11): p. 2448-2450.

De Bruin, E.D., et al., Quantification of everyday motor function in a geriatric population. J Rehabil Res Dev, 2007. 44(3): p. 417-28.

Najafi, B., et al., Measurement of stand-sit and sit-stand transitions using a miniature gyroscope and its application in fall risk evaluation in the elderly. Ieee Transactions on Biomedical Engineering, 2002. 49(8): p. 843-851.

Aminian, K., et al., Evaluation of an ambulatory system for gait analysis in hip osteoarthritis and after total hip replacement. Gait & Posture, 2004. 20(1): p. 102-107.

Kempen, G. I., L. Yardley, et al. (2008). The Short FES-I: a shortened version of the falls efficacy scale-international to assess fear of falling. Age Ageing 37(1): 45-50.

Gandek, B., J. E. Ware, et al. (1998). Cross-validation of item selection and scoring for the SF-12 Health Survey in nine countries: results from the IQOLA Project. International Quality of Life Assessment. J Clin Epidemiol 51(11): 1171-1178.

Guralnik, J. M., E. M. Simonsick, et al. (1994). A short physical performance battery assessing lower extremity function: association with self-reported disability and prediction of mortality and nursing home admission. J Gerontol 49(2): M85-94.

31. Grewal, G. Sayeed R, Schwenk M, Bharara M, Menzies R, Talal K, Armstrong D, Najafi B, ’Balance Rehabilitation – Promoting the Role of Virtual Reality in Patients with [Diabetic Peripheral Neuropathy](http://surgery.arizona.edu/unit/center/iCAMP/publications), Journal of American Podiatric Medicine Association (JAPMA), 2013, [Accepted for Publication].
