# Supplementary material for: A Pilot Clinical Trial to Objectively Assess the Efficacy of Electroacupuncture on Gait in Patients with Parkinson's Disease Using Body Worn Sensors
Source: PLoS One. 2016 May 26;11(5):e0155613. doi: 10.1371/journal.pone.0155613 (PMC4882016; doi:10.1371/journal.pone.0155613)
Supplement: S1 Table — (DOCX) [file pone.0155613.s005.docx]

S1 Table: Comparison of responses to treatment between experimental and control groups

| **Parameter** | **Experimental**  **Mean (SD) Changes** | | **Control**  **Mean (SD) Changes** | **95% CI**^&^ | | ***p*-value** | **Effect Size** |
| --- | --- | --- | --- | --- | --- | --- | --- |
| Speed-STHW | | 0.07 (0.11) ↑ | 0.03 (0.14) ↑ | 0.03 | 0.06 | 0.001* | 0.32 |
| Speed-DTHW | | 0.08 (0.19) ↑ | 0.01 (0.10) ↑ | 0.05 | 0.08 | 0.001* | 0.67 |
| Speed-STFW | | 0.13 (0.14) ↑ | 0.01 (0.10) ↑ | 0.08 | 0.14 | 0.001* | 0.99 |
| Speed-DTFW | | 0.21 (0.22) ↑ | 0.02 (0.07) ↑ | 0.10 | 0.27 | 0.001* | 1.16 |
| Stride-STHW | | 0.05 (0.06) ↑ | 0.04 (0.08) ↑ | -0.02 | 0.04 | 0.630 | 0.14 |
| Stride-DTHW | | 0.03 (0.10) ↑ | 0.09 (0.15) ↑ | -0.10 | 0.04 | 0.199 | 0.47 |
| Stride-STFW | | 0.06 (0.08) ↑ | 0.01 (0.09) ↑ | 0.02 | 0.06 | 0.072 | 0.59 |
| Stride-DTFW | | 0.10 (0.14) ↑ | 0.01 (0.06) ↑ | 0.06 | 0.13 | 0.001* | 0.84 |
| Cadence-STHW | | 1.82 (3.88) ↑ | -0.86 (6.92)↓ | 1.62 | 4.17 | 0.001* | 0.48 |
| Cadence-DTHW | | 3.08 (7.15) ↑ | 1.64 (5.12) ↑ | -1.25 | 5.11 | 0.636 | 0.23 |
| Cadence-STFW | | 2.59 (5.89) ↑ | -0.86 (2.30)↑ | 2.45 | 4.50 | 0.001* | 0.77 |
| Cadence-DTFW | | 5.30 (6.28) ↑ | 1.86 (2.99) ↑ | 0.64 | 6.24 | 0.023* | 0.70 |
| Double support-STHW | | -0.64(1.85) ↓ | 0.32 (3.13) ↑ | -1.98 | -0.02 | 0.103 | 0.37 |
| Double support-DTHW | | -1.15(2.89) ↓ | -1.27 (4.38) ↓ | -2.65 | 2.45 | 0.853 | 0.03 |
| Double support-STFW | | -2.08 (3.04) ↓ | -0.56 (2.25) ↓ | -2.46 | -3.15 | 0.001* | 0.57 |
| Double support-DTFW | | -2.03 (2.73) ↓ | -2.55 (3.68) ↓ | -1.40 | 2.10 | 0.689 | 0.16 |
| Midswing -STHW | | 14.3 (23.6) ↑ | 11.3 (38.1) ↑ | -4.92 | 13.0 | 0.566 | 0.37 |
| Midswing -DTHW | | 10.1 (51.1) ↑ | 29.8 (55.3) ↑ | -38.4 | 9.24 | 0.304 | 0.37 |
| Midswing -STFW | | 21.5 (38.0) ↑ | 17.2(16.7) ↑ | -0.94 | 11.8 | 0.167 | 0.15 |
| Midswing -DTFW | | 40.1(41.5) ↑ | 21.5 (18.7) ↑ | 3.21 | 34.0 | 0.023* | 0.58 |
| SF-12 (PCS) | | 0.6 (6.40) ↑ | 1.0 (7.5) ↓ | -10.8 | 7.8 | 0.71 | 0.23 |
| SF-12 (MCS) | | 3.7 (11.20) ↑ | 0.5 (3.4) ↑ | -11.6 | 5.2 | 0.42 | 0.39 |
| FES-I | | 2.2 (3.90) ↓ | 1.8 (3.8) ↑ | -0.8 | 8.8 | 0.09 | 1.04 |
| VAS | | 1.1 (3.00) ↓ | 1.2 (2.8) ↓ | -3.6 | 3.4 | 0.95 | 0.03 |
| UPDRS part I | | 2.6 (2.10) ↓ | 1.8 (2.7) ↑ | -7.6 | -1.1 | 0.005* | 1.82 |
| UPDRS part II | | 7.2 (5.50) ↓ | 0.4 (3.4) ↓ | -5.9 | -0.5 | 0.02* | 1.49 |
| UPDRS part III | | 16.0 (6.20) ↓ | 3.0 (5.2) ↑ | -13.2 | -5.7 | <.001* | 3.30 |

STHW=single-task habitual walking; DTHW=dual-task habitual walking; STFW=single-task fast walking; DTFW= dual-task fast walking; SF-12 (PCS)=Short Form-12 Health Survey (physical component summary); SF-12 (MCS) =Short Form-12 Health Survey (mental component summary); FES-I =short Falls Efficacy Scale-International; VAS=visual analog scale for pain. UPDRS = Unified Parkinson's disease Rating Scale. The symbol * indicates a significant difference between groups after treatment (Bootstrap for Independent Sample Test, *p*<0.05). CI^&^: Bootstrap Confidence Intervals. Symbol ↓ indicates a reduction and ↑ an increase in each parameter following the treatment. Cohen’s d was used for calculating effect size.
