# Supplementary material for: A Pilot Clinical Trial to Objectively Assess the Efficacy of Electroacupuncture on Gait in Patients with Parkinson's Disease Using Body Worn Sensors
Source: PLoS One. 2016 May 26;11(5):e0155613. doi: 10.1371/journal.pone.0155613 (PMC4882016; doi:10.1371/journal.pone.0155613)
Supplement: S2 Table — (DOCX) [file pone.0155613.s006.docx]

S2 Table: Comparison of responses to treatment within experimental and control groups in subjective reports and UPDRS

| **Parameter** |  | **Experimental** | | | | | | |  | | **Control** | | | | | | | |
| --- | --- | --- | --- | --- | --- | --- | --- | --- | --- | --- | --- | --- | --- | --- | --- | --- | --- | --- |
|  |  | **Pre-EA**  **Mean (SD)** | **Post-EA**  **Mean(SD)** | **% change** | **95% CI** | | ***p*-value** | | |  | **Pre-EA**  **Mean (SD)** | **Post-EA**  **Mean(SD)** | **% change** | | | **95% CI** | | ***p*-**  **value** |
| SF-12 (PCS) |  | 39.7 (11.7) | 40.3 (14.1) | 1% ↑ | -11.6 | 12.8 | | 0.82 | |  | 34.7 (9.4) | 33.7 (5.3) | | 3% ↓ | -12.6 | | 10.7 | 0.85 |
| SF-12 (MCS) |  | 43.4 (12.6) | 47.1 (8.5) | 8% ↑ | -6.5 | 13.9 | | 0.45 | |  | 47.2 (7.3) | 47.7 (7.9) | | 1% ↑ | -10.7 | | 11.6 | 0.93 |
| FES-I |  | 17.2 (6.8) | 14.6 (6.9) | 15% ↓ | -9.0 | 3.8 | | 0.41 | |  | 16.6 (5.0) | 18.4 (4.0) | | 11% ↑ | -4.9 | | 8.5 | 0.55 |
| VAS |  | 2.5 (3.2) | 1.4 (2.4) | 44% ↓ | -3.7 | 1.5 | | 0.26 | |  | 3.4 (3.3) | 2.2 (3.0) | | 35% ↓ | -5.8 | | 3.4 | 0.50 |
| UPDRS-part I |  | 5.3 (3.5) | 2.7 (2.3) | 49% ↓ | -5.5 | 0.6 | | <0.01* | |  | 3.3 (2.4) | 5.1 (4.2) | | 55% ↑ | -3.4 | | 7.0 | 0.21 |
| UPDRS-part II |  | 18.0 (9.7) | 10.8 (6.6) | 46% ↓ | -10.6 | -3.8 | | <.0001* | |  | 17.0 (5.7) | 16.6 (7.9) | | 9% ↑ | -3.3 | | 2.5 | 0.80 |
| UPDRS-Part III |  | 35.1 (15.3) | 19.1 (10.9) | 40% ↓ | -12.1 | -19.8 | | <.01* | |  | 34.2 (12.3) | 37.2 (11.6) | | 2% ↑ | -1.6 | | 7.6 | 0.27 |

SF-12 (PCS) = Short Form-12 Health Survey (physical component summary); SF-12 (MCS) = Short Form-12 Health Survey (mental component summary); FES-I =short Falls Efficacy Scale-International; VAS=visual analog scale for pain. UPDRS = Unified Parkinson's disease Rating Scale.CI: Confidence Intervals; The symbol * indicates a significant difference (t-test, *p*<0.05). Symbol ↓ indicates a reduction and ↑ an increase in each parameter following the treatment.
